# VA-ECMO and IABP Supported CHIP-PCI for Left Main Bifurcation and CTO After Failed CABG

**DOI:** 10.1016/j.jaccas.2026.109017

**Published:** 2026-07-29

**Authors:** Pedro Henrique Ferro de Brito, André Moreira Nicolau, Samuel Padovani Steffen, Alexandre Antônio da Cunha Abizaid, Fábio Sândoli de Brito Júnior, Carlos M. Campos

**Affiliations:** Instituto do Coração da Faculdade de Medicina da Universidade de São Paulo (InCor/HCFMUSP), São Paulo, Brazil

**Keywords:** bifurcation PCI, calcified coronary lesions, chronic total occlusion (CTO), complex high-risk indicated PCI (CHIP), ischemic cardiomyopathy, left main coronary artery, mechanical circulatory support

## Abstract

**Background:**

Patients with advanced coronary artery disease, prior coronary artery bypass grafting failure, and left ventricular dysfunction represent a particularly high-risk population, especially in last remaining vessel scenarios. Complex percutaneous coronary intervention (PCI) involving left main bifurcation disease, chronic total occlusions, and severe calcification carries substantial risk of hemodynamic collapse.

**Case Summary:**

A 66-year-old man with prior coronary artery bypass grafting and ischemic cardiomyopathy presented with limiting angina and dyspnea. Coronary angiography demonstrated occluded left internal mammary artery graft, severely degenerated saphenous vein graft, chronic total occlusions of the circumflex and right coronary arteries, severe left main disease, and diffuse calcified disease of the left anterior descending artery. After heart team discussion, the patient was deemed inoperable. Complex high-risk indicated PCI was successfully performed under combined venoarterial extracorporeal membrane oxygenation and intra-aortic balloon pump support.

**Discussion:**

Prophylactic mechanical circulatory support may provide hemodynamic stability and facilitate safe, complete revascularization in extreme high-risk coronary interventions.

**Take-Home Message:**

In inoperable patients with advanced coronary disease and ventricular dysfunction, mechanically supported complex high-risk indicated PCI may enable safe and effective complete revascularization.

## Visual Summary

**Figure:**
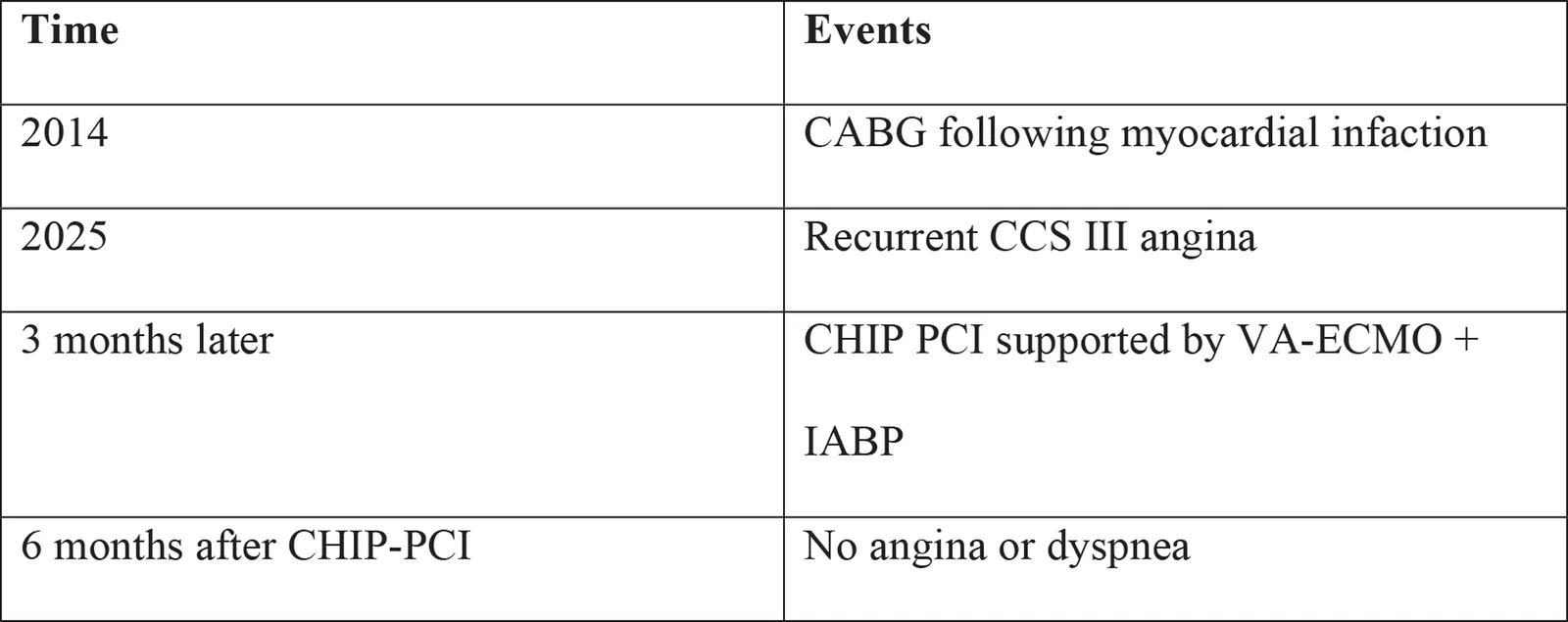

## History of Presentation

**Figure 1:**
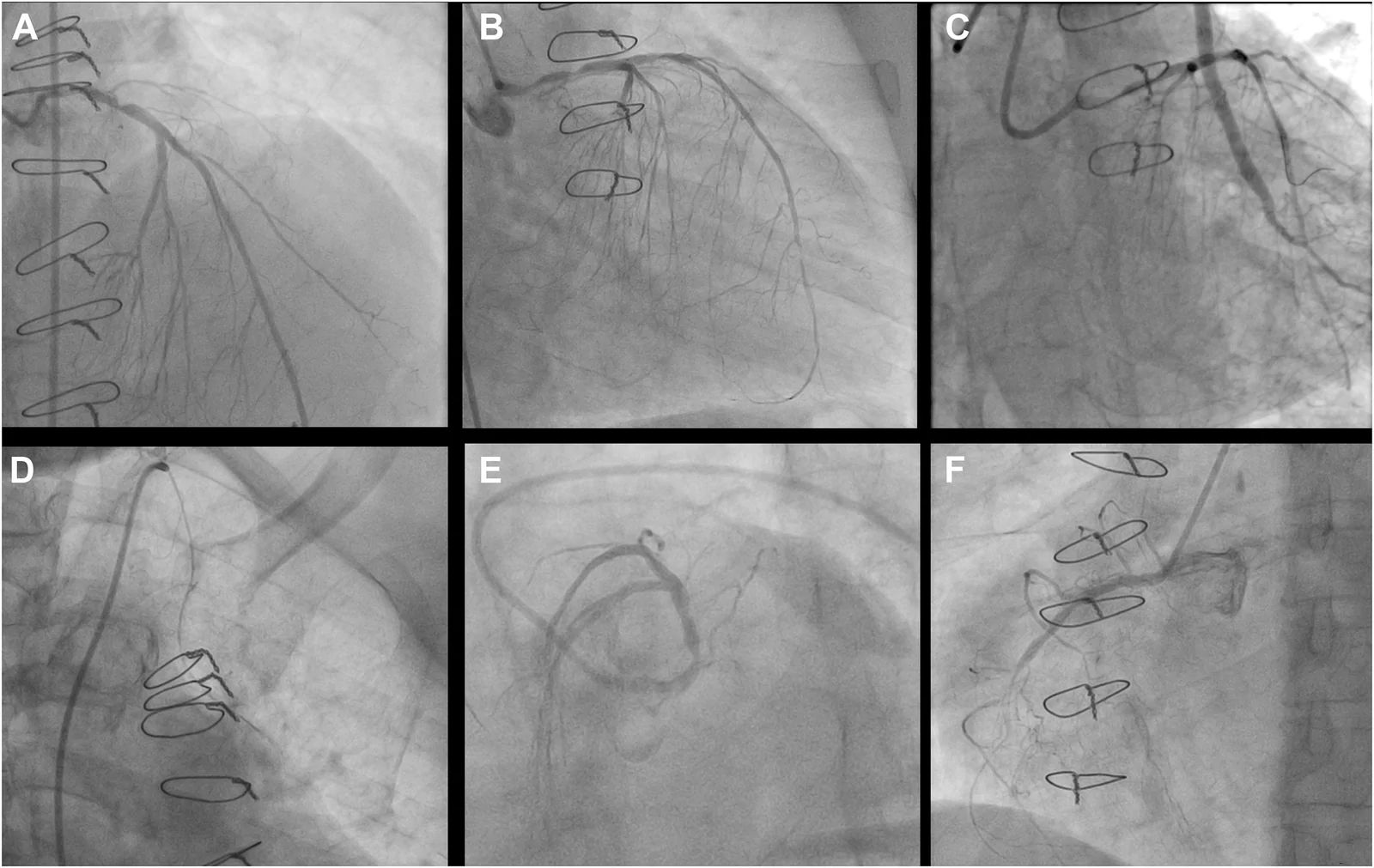
Baseline Coronary Anatomy and Graft Assessment (A and B) Severe left main coronary artery (LMCA) disease involving the ostial and proximal left anterior descending (LAD) artery, with concomitant chronic total occlusion (CTO) of the left circumflex (LCx). (C) Severe stenosis at the distal anastomosis of the saphenous vein graft to the obtuse marginal branch, with the absence of retrograde collateral flow from the obtuse marginal territory to the LCx. (D) Occluded left internal mammary artery graft. (E) Severe LMCA and proximal LAD disease in a spider view, associated with CTO of the LCx. (F) Chronic total occlusion of the right coronary artery, consistent with advanced multivessel coronary artery disease.

A 66-year-old man presented in the outpatient setting with recurrent angina (Canadian Cardiovascular Society class III) and dyspnea (NYHA functional class II). Given his history of prior coronary artery bypass grafting and worsening symptoms, coronary angiography was performed, demonstrating chronic total occlusions (CTOs) of the right coronary and left circumflex arteries (LCx), severe left main disease, and severe ostial and proximal left anterior descending artery (LAD) disease. The left internal mammary artery graft was proximally occluded, and the saphenous vein graft to the second obtuse marginal branch showed a severely diseased distal anastomosis, consistent with an advanced graft failure scenario (Figure 1).

The case was discussed by the heart team, and percutaneous coronary intervention was selected as the revascularization strategy despite a complex high-risk indicated percutaneous coronary intervention (CHIP) score of 11, given the prohibitive surgical risk associated with redo coronary artery bypass grafting in the setting of severe left ventricular dysfunction and multiple comorbidities.

## Past Medical History

The patient had a history of heart failure with reduced ejection fraction (left ventricular ejection fraction 31%), prior coronary artery bypass grafting in 2014 after myocardial infarction, ischemic stroke in 2023, type 2 diabetes mellitus, hypertension, and severe peripheral arterial disease with bilateral occlusion of the anterior tibial and right radial arteries. He was also a former smoker.

## Investigations

Coronary angiography was performed to define the extent and severity of coronary artery disease. In addition, a peripheral arterial ultrasound was obtained to guide vascular access planning, given the presence of severe peripheral arterial disease.

## Management

Following heart team consensus to proceed with percutaneous revascularization, vascular ultrasound was performed to guide access strategy. An option combining venoarterial extracorporeal membrane oxygenation (VA-ECMO) and intra-aortic balloon pump (IABP) was adopted to simultaneously support cardiac output and reduce the afterload increase caused by VA-ECMO (left ventricular venting and unloading). An intra-aortic balloon pump (IABP) was inserted via the right femoral artery, which presented as the worst peripheral arterial disease among the femoral vessels. VA-ECMO was established via the left femoral artery and vein.

The left radial artery was cannulated with a 6-F sheath for potential simultaneous injection via the saphenous vein graft. Because of right radial artery occlusion, the right brachial artery was accessed, and a 7-F XB guiding catheter (Cordis) was advanced to engage the native left coronary system. Angiography of the saphenous vein graft to the second obtuse marginal branch demonstrated patency limited to the distal vessel beyond the anastomosis, without retrograde filling of the LCx. Given the absence of retrograde flow and the presence of a CTO of the right coronary artery, a simultaneous injection strategy was abandoned.

During initial instrumentation of the left main coronary artery (LMCA), the patient developed sustained ventricular tachycardia with preserved perfusion, without hemodynamic compromise due to ongoing mechanical circulatory support. A 0.014-inch Sion Black guidewire (Asahi Intecc) was advanced into the distal LAD, followed by disengagement of the guiding catheter, resulting in prompt restoration of sinus rhythm.

Attention was then directed to the LCx CTO. A Caravel microcatheter (Asahi Intecc) was advanced over a 0.014-inch BMU wire (Abbott Vascular), which was subsequently exchanged for a Fielder XTA (Asahi Intecc) and then a Gaia Next 2 (Asahi Intecc) wire using an antegrade wire escalation strategy. Successful CTO crossing was achieved with the Gaia Next 2 wire, allowing distal positioning of the microcatheter and exchange to a workhorse wire. Lesion preparation of the LCx was performed with sequential balloon dilatation (Mini Trek 2.0 × 20 mm and Trek 2.5 × 20 mm; Abbott Vascular), followed by calcium modification using a Wolverine cutting balloon (Boston Scientific) (3.0 × 10 mm, high-pressure inflations) and rotablator rotational atherectomy system (Boston Scientific) with a 1.5-mm burr.

Subsequently, rotational atherectomy of the LMCA-LAD segment was performed with a 1.75-mm burr after wire exchange to a Rotawire, and further lesion preparation was achieved with high-pressure noncompliant balloon dilatation (NC Trek Neo 3.5 × 15 mm; Abbott Vascular).

**Figure 2:**
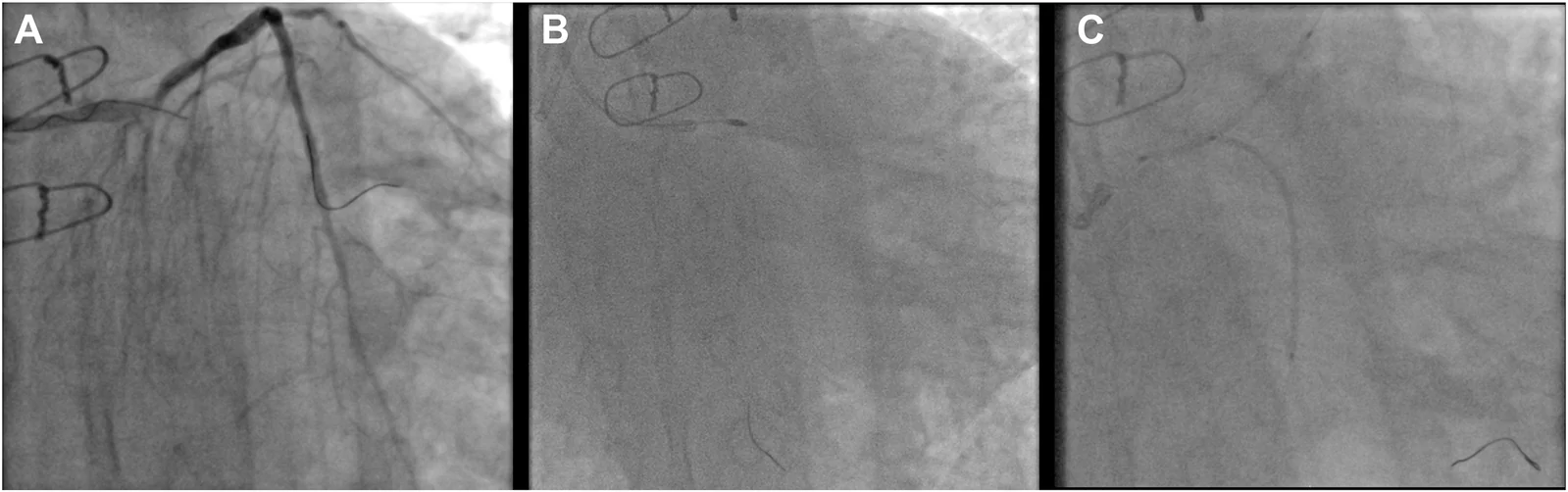
Complex Percutaneous Coronary Intervention Strategy With Calcium Modification and Bifurcation Stenting (A) Dual wiring of the left anterior descending artery and left circumflex (LCx) with attempted chronic total occlusion crossing of the LCx. (B) Rotational atherectomy performed for calcium modification of the left main bifurcation. (C) Stent positioning according to a planned mini-crush technique at the left main coronary artery bifurcation.

Left main bifurcation stenting was then performed using a mini-crush technique, with deployment of a 3.0 × 38-mm Promus Elite stent (Boston Scientific) in the LCx and a 4.0 × 38-mm Xience Alpine stent (Abbott Vascular) from the LMCA to the LAD. The proximal optimization technique was performed with a 4.5 × 12-mm noncompliant balloon at high pressure, followed by rewiring of the LCx and final kissing balloon inflation using two 3.5 × 15-mm noncompliant balloons (Figure 2).

**Figure 3:**
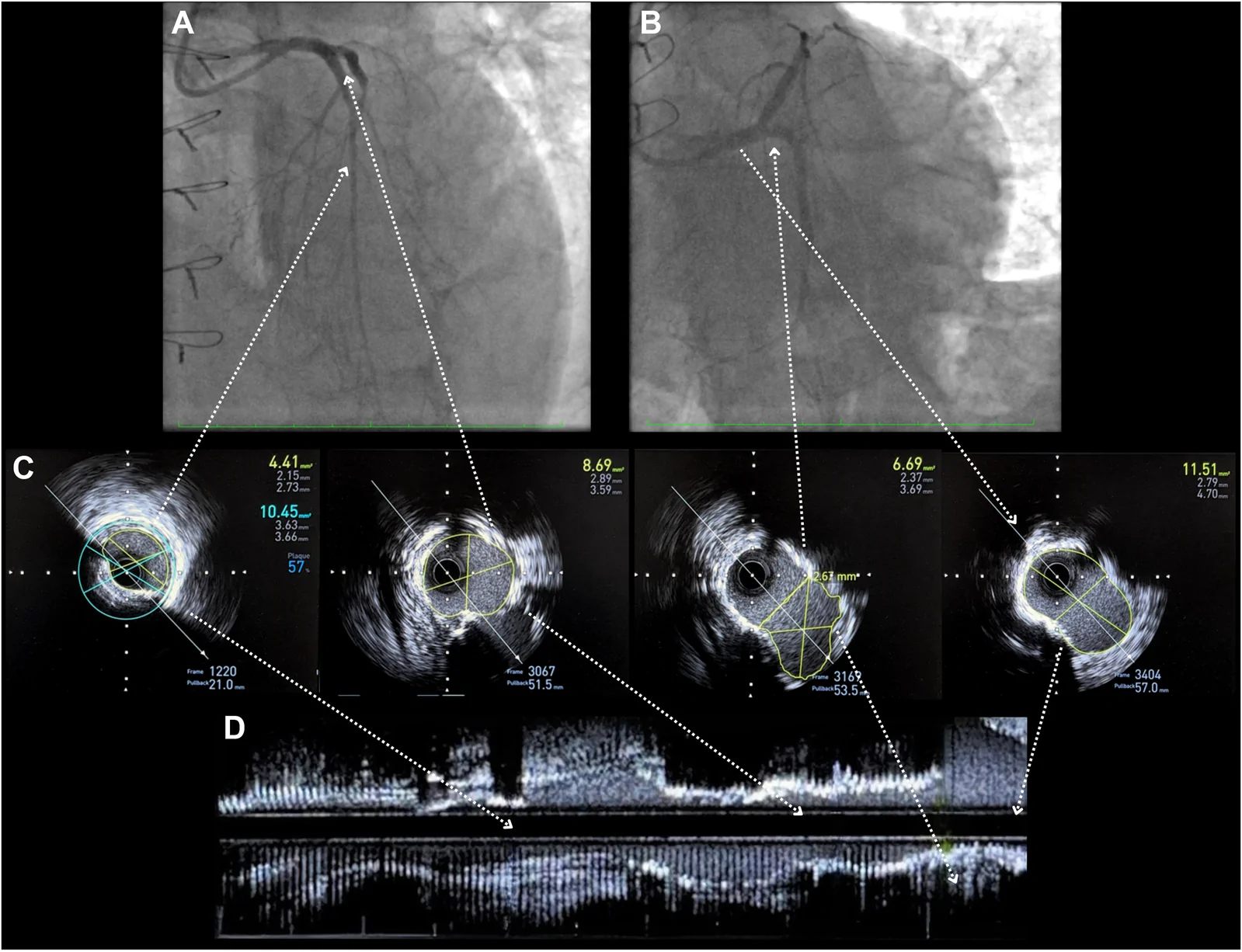
IVUS Assessment and Final Results (A) IVUS demonstrating an intermediate lesion in the mid–left anterior descending (LAD) artery, which was deferred and managed conservatively. (B) In-stent minimal luminal area of the LAD, confirming adequate stent expansion, with the corresponding final angiographic result. (C) IVUS assessment of the left circumflex ostium showing the luminal area and diameter after intervention. (D) Final IVUS of the left main coronary artery demonstrating satisfactory in-stent expansion and the optimal procedural result. IVUS = intravascular ultrasound.

Final intravascular ultrasound assessment (OptiCross 40 MHz; Boston Scientific) of the LAD demonstrated excellent stent apposition and expansion, with an expansion index of 83% and a minimal luminal area of 8.69 mm^2^. The LCx ostium demonstrated an optimal result as determined by the OCTOBER trial criteria,^1^ with a mean ostial diameter of 3.03 mm. An additional angiographically intermediate lesion in the mid-LAD was assessed by intravascular ultrasound, showing a minimal luminal area of 4.41 mm^2^ and a plaque burden of 57%, and was therefore managed conservatively (Figure 3).

## Outcome and Follow-Up

The patient was successfully decannulated from VA-ECMO in the hybrid operating room immediately after the procedure. The IABP was removed 36 hours later. He was discharged after 8 days, having recovered from procedure-related acute kidney injury, with a creatinine level of 1.16 mg/dL at discharge.

At 6-month follow-up, the patient remained free of angina and dyspnea, with significant functional improvement. He reported intermittent claudication, likely reflecting the underlying peripheral arterial disease, which became more clinically apparent after improvement in functional capacity. Follow-up transthoracic echocardiography demonstrated improvement in left ventricular ejection fraction from 31% to 40%. Renal function at 6 months showed a creatinine level of 1.76 mg/dL, corresponding to an estimated glomerular filtration rate of 42 mL/min/1.73 m^2^.

## Discussion

Percutaneous revascularization of the LMCA in patients with left ventricular dysfunction represents a prognostically meaningful intervention, as it restores perfusion to a large myocardial territory and may improve survival and ventricular function recovery.^2^^,^^3^ This benefit is particularly relevant in the setting of a “last remaining patent vessel,” where severe LMCA disease places the patient at high risk of extensive myocardial ischemia and sudden hemodynamic collapse. These features define a CHIP patient, as characterized by contemporary risk stratification models developed to identify patients at increased procedural risk and guide the use of adjunctive support strategies.^4^

In this context, LMCA PCI is inherently high risk, especially when associated with complex anatomy such as bifurcation lesions, CTOs, and severe calcification requiring advanced lesion preparation.^2^^,^^3^^,^^5^^,^^6^ Transient interruption of coronary flow during the procedure may result in rapid hemodynamic deterioration, particularly in patients with limited ventricular reserve.

In such scenarios, prophylactic use of high-flow mechanical circulatory support is critical. Devices such as VA-ECMO provide full cardiopulmonary support, ensuring hemodynamic stability and end-organ perfusion during complex interventions.^7^, ^8^, ^9^ In the present case, during initial instrumentation of the LMCA, the patient developed ventricular tachycardia without hemodynamic compromise, underscoring the protective role of preemptive mechanical circulatory support. This support enables operators to safely perform advanced lesion preparation and optimization techniques, facilitating complete revascularization in patients otherwise deemed inoperable. However, VA-ECMO increases cardiac afterload; therefore, combining it with an IABP may mitigate left ventricular distension and improve coronary perfusion, representing a complementary and synergistic strategy. The role of mechanical circulatory support in high-risk PCI has been explored in randomized data such as the BCIS-1 trial.^10^

Importantly, in resource-limited health care systems such as the Brazilian public health system (SUS), advanced percutaneous ventricular assist devices such as Impella are not widely available. In this context, the combined use of VA-ECMO and IABP represents a pragmatic and potentially more accessible alternative, enabling the safe performance of complex high-risk PCI procedures that would otherwise be unfeasible (Table 1). This strategy may expand access to advanced revascularization therapies in public health care settings while maintaining procedural safety in critically high-risk patients.

**Table 1:** Comparison of Mechanical Circulatory Support Devices for High-Risk PCI (IABP, Impella CP, VA-ECMO, and VA-ECMO Plus IABP)

Feature	IABP	Impella CP	VA-ECMO	VA-ECMO + IABP
Primary hemodynamic goal	Afterload reduction and diastolic augmentation	Active LV unloading and systemic support	Full circulatory and respiratory support	Full support + active LV unloading (venting)
Cardiac output support	Modest (approximately 0.5-1.0 L/min)	Significant (up to 4.3 L/min)	Up to 7.0 L/min	Up to 7.0 L/min
Afterload impact	Decreases (via rapid deflation)	Decreases (via active LV drainage)	Increases (retrograde aortic flow)	Neutral/decreased (IABP offsets ECMO afterload)
Vascular access	7.5-F to 8-F (femoral)	14-F (femoral)	15-F to 22-F art/19-F to 25-F ven	Dual access (multivessel)
Approximate cost (disposable kit only)	$	$$$$	$$	$$/$$$
Common complications	Balloon rupture, limb ischemia (rare), and thrombocytopenia	Hemolysis, limb ischemia, device migration, and bleeding	LV distension, stroke, limb ischemia, and bleeding	Higher bleeding risk, vascular injury, and infection

Dollar-sign symbols indicate increasing relative cost, from the lowest tier (one symbol) to the highest tier (four symbols); absolute values vary by country.

CABG = coronary artery bypass grafting; CP = cardiac power (Impella CP, Abiomed); LV = left ventricular; IABP = intra-aortic balloon pump; VA-ECMO = venoarterial extracorporeal membrane oxygenation.

## Conclusions

In patients with advanced coronary artery disease, left ventricular dysfunction, and failed surgical revascularization, left main bifurcation PCI may represent the only viable and prognostically meaningful treatment option. In this high-risk setting—particularly in the presence of a last remaining patent vessel—the use of prophylactic high-flow mechanical circulatory support may be essential in selected cases to maintain hemodynamic stability and enable safe, complete revascularization.

**Take-Home Message**

• In inoperable patients with advanced coronary disease and ventricular dysfunction, complex high-risk indicated percutaneous coronary intervention supported by mechanical circulatory assistance may allow safe and effective revascularization.

## Funding support and author disclosures

The authors have reported that they have no relationships relevant to the contents of this paper to disclose.

**Visual Summary:** Timeline of the Case

Time	Events
2014	CABG after myocardial infaction
2025	Recurrent CCS III angina
3 months later	CHIP-PCI supported by VA-ECMO + IABP
6 months after CHIP-PCI	No angina or dyspnea

CABG = coronary artery bypass grafting; CCS III = Canadian Cardiovascular Society III; CHIP-PCI = complex high-risk indicated percutaneous coronary intervention; IABP = intra-aortic balloon pump; VA-ECMO = venoarterial extracorporeal membrane oxygenation.
